# How slow K^+ ^currents impact on spike generation mechanism?

**DOI:** 10.1186/1471-2202-16-S1-P125

**Published:** 2015-12-18

**Authors:** Ryota Kobayashi, Katsunori Kitano

**Affiliations:** 1 Principles of Informatics Research Division, National Institute of Informatics, 2-1-2 Hitotsubashi, Chiyoda-ku, Tokyo, Japan; 2 Department of Informatics, SOKENDAI (The Graduate University for Advanced Studies), 2-1-2 Hitotsubashi, Chiyoda-ku, Tokyo, Japan; 3 Department of Human and Computer Intelligence, Ritsumeikan University, 1-1-1 Nojihigashi, Kusatsu, Shiga 525-8577, Japan

## 

Neuronal adaptation is the change in the responsiveness of a neuron over time, and may improve coding information from an environment. Adaptation originates from various factors, including single neurons, synapses, and network dynamics. Here we investigate adaptation in a responsiveness of a neuron. When a neuron received prolonged stimulation, it initially responds with a high firing rate, and the firing rate decrease. This is called spike-frequency adaptation, which is observed in most pyramidal neurons in various animals. Spike-frequency adaptation is usually accounted for by slow K^+ ^currents, for example, the M-type K^+ ^current (*I*_M_) and the Ca^2+^-activated K^+ ^current (*I*_AHP_), and the conductance-based (Hodgkin−Huxley type) models including the slow K^+ ^currents have succeeded to reproduce the electro-physiological properties of a neuron [1].

The detailed biophysical mechanism underlying spike-frequency adaptation may impact on the coding property of a neuron [2,3]. For example, it was suggested that *I*_M _facilitates the spike-timing coding, whereas *I*_AHP _improves the spike rate-coding [2] and *I*_M _increases the response to low-frequency input signals, whereas *I*_AHP _decreases the response to low-frequency signals [3].

Due to the complexity of the conductance-based models, it is not clear how the slow K^+ ^currents impact on spike generation mechanism, more specifically, how the parameters of the slow K^+ ^currents regulate spike generation. For understanding the impact of slow K^+ ^currents, we have developed a framework to reduce a detailed conductance-based model with slow K^+ ^currents to an adaptive threshold model [4]. We have deduced a formula that links the slow K^+ ^parameters to the parameters of the reduced model. The formula was validated with the simulation of the detailed model. This formula clarifies how *I*_M _and *I*_AHP _impact on spike generation mechanism differently and the parameters of *I*_M _and *I*_AHP _influence spike generation.
